# Supplementary material for: Integrated Comparative Transcriptome and circRNA-lncRNA-miRNA-mRNA ceRNA Regulatory Network Analyses Identify Molecular Mechanisms Associated with Intramuscular Fat Content in Beef Cattle
Source: Animals (Basel). 2023 Aug 11;13(16):2598. doi: 10.3390/ani13162598 (PMC10451991; doi:10.3390/ani13162598)
Supplement: Supplementary file 1 [file animals-13-02598-s001.zip › Supplementary Material 6.pdf]

Table S6. Information about differentially expressed types of RNAs and their interactions in subnets of ceRNA regulatory network between eight pairwise comparative groups associated with IMF in beef cattle breeds.

Subnets	Important Interactions				Types of Hub RNAs: mRNAs Down/Up Regulation (↓↑)	Types of Hub RNAs: miRNAs Down/Up Regulation (↓↑)	Types of Hub RNAs: lncRNAs Down/Up Regulation (↓↑)
1	mRNA-miRNA	bta-miR-1-2	Suppressed	UTRN, ACTB, ASH1L, NRP1, THBS1	(↓) UTRN, ATP2A2, CTNND1, MYOZ1, CKM, LMOD2, BAG3 (↑) EHBP1L1	(↓) bta-miR-1-2	(NA) MSTRG.10337
	mRNA-lncRNA	LOC512486	Interacted with	IRF1			
		MSTRG.2819	Interacted with	MYPN			
		MSTRG.10337	Interacted with	RYR1, CASQ1, MYBPC1, TNNT1, CACNA1S, CKM, LMOD2, CORO6, MB, MYOM2, LDB3			
	lncRNA-lncRNA	MSTRG.10337	Interacted with	MSTRG.2819, MSTRG.5283, MSTRG.8140, MSTRG.8974, MSTRG.8142, MSTRG.7965			
2	mRNA-miRNA	bta-miR-1-2	Suppressed	UTRN, THBS1, ASH1L HNRNPA1, NRP1, ACTB, FN1	(↓) UTRN, MYOZ1, RYR1, LMOD2, CKM (↑) EHBP1L1, MSN	(↓) bta-miR-1-2	(NA) MSTRG.10337
		bta-miR-133a-1, bta-miR-133a-2	Suppressed	MSN			
	mRNA-lncRNA	MSTRG.10337	Interacted with	LMOD2, MYOM2, LDB3, RYR1, NEB, MYBPC1, TNNC2, MYL2, CORO6, CKM, MB, TNNT1, FBXO40			
		LOC526226, LOC101903367, LOC112444238, LOC525506, LOC525506	Interacted with	MEF2C, IGF2			
		BIANCR	Interacted with	S100B, HSPA1A, ACTR3B, HADHB			
3	mRNA-miRNA	bta-miR-1-2	Suppressed	UTRN, THBS1, ASH1L HNRNPA1, NRP1, ACTB, FN1	(↓) ATP2A2, MYOZ1, RYR1, DNAJB4, BIRC6, BAG3 (↑) EHBP1L1	(↓) bta-miR-365-1	(NA) BIANCR
		bta-miR-95	Suppressed	ACACB, ENAH, IPO5, XPO4, LPIN1			
		bta-miR-365-1	Suppressed	PTGFRN, CCNG1, ASH1L, BRWD1, SARS, UBR3, YWHAG, CLUH, PKIA, EIF4EBP2, HSPB8, NOTCH3, ADCY9, VAPA, ATP1A2, IPO5, ENAH, CD180, ACACB, EHD3, ARHGAP35, PPP1R2, BCL2L13, CD38, CD74, TAL1, ACP5, DST, APC			
	mRNA-lncRNA	BIANCR	Interacted with	ACTR3B, MAP3K11, NDRG2, LPIN1			

4	mRNA-miRNA	bta-miR-133a-2, bta-miR-133a-1	Suppressed	ASH1L, XPO4	(↓) GLUL, BRWD1, ASH1L, VAPS13D, BIRC6, VAPS13C, WDFY3	(↓) bta-miR-1296, bta-miR-378d	**(NA)** BIANCR
		bta-miR-95	Suppressed	IPO5, ABLIM1			
		bta-miR-1-2	Suppressed	KCNJ2, HIPK3			
		bta-miR-378d	Suppressed	CYB5R1, FYTTD1, AFF1, FXR1, NCKAP1, CLUH, MORF4L2, CIITA, PDE4D, VAPA, RTN2, MAPRE3, HIPK3, KCNJ2, PRR5L, TMEM151A, WARS, SDS, CLIC5, GSTM4, DPYSL3, DPYSL3, FBXO40			
		bta-miR-1296	Suppressed	TNNI1, C3, ABLIM1, WNK1, ADCY9, ZNF106, VPS13C, PKIA, CARM1, EIF4EBP2, AOC3			
	mRNA-circRNA	circRNA5689, circRNA5807, circRNA5797, circRNA5755	Interacted with	GLUL			
	mRNA-lncRNA	MSTRG.3939.1, MSTRG.21819.1, MSTRG.3767.1, MSTRG.20646.1, MSTRG.20716.2, MSTRG.19454.1, MSTRG.7800.1, MSTRG.14115.1, MSTRG.10285.2, MSTRG.19644.1, MSTRG.5117.1, MSTRG.5458.1, MSTRG.11792.1, MSTRG.16187.2, MSTRG.10293.1, MSTRG.10451.1, MSTRG.18715.1, MSTRG.16614.1, MSTRG.22368.1, MSTRG.6618.1, MSTRG.20293.2, MSTRG.22366.1, MSTRG.7874.1, MSTRG.12913.1, MSTRG.20966.1, MSTRG.22369.1, MSTRG.9461.1, MSTRG.16045.1, MSTRG.8171.1, MSTRG.7718.1, BIANCR	Interacted with	GLUL			
		BIANCR	Interacted with	S100B			
	miRNA-circRNA	bta_circ_0004158, bta_circ_0006814, bta_circ_0006815, bta_circ_0006807, bta_circ_0006152, bta_circ_0008914, bta_circ_0008835, bta_circ_0006806, bta_circ_0006813	Interacted with	bta-miR-378d			

		bta_circ_0006812, bta_circ_0010653, bta_circ_0003169	Interacted with	bta-miR-1296			
5 and 6	mRNA-miRNA	bta-miR-133a-2, bta-miR-133a-1, bta-miR-1-2	Suppressed	ASH1L	(🡻) BRWD1, ASH1L, VAPS13D, BIRC6, VAPS13C, WDFY3	(🡻) bta-miR-1296	-
		bta-miR-1296	Suppressed	CD180, ABLIM1, RABEP1, EIF4EBP2, TNNI1, AOC3, C3, WNK1, ADCY9, EHD3, PKIA, CARM1, ZNF106, CSDE1			
	miRNA-circRNA	bta_circ_0010653, bta_circ_0003169	Interacted with	bta-miR-1296			
7	mRNA-miRNA	bta-miR-378d	Suppressed	RTN2, DPYSL3, PDE4D, KCNJ2, CYB5R1, GSTM4, FYTTD1, CLIC5, MORF4L2, EHD3, CD180, PRR5L, TMEM151A, CLUH, AFF1, CIITA, VAPA	(🡻) RTN2	(🡻) bta-miR-378d	-
	miRNA-circRNA	bta-miR-378d	Interacted with	bta_circ_0006813, bta_circ_0006814, bta_circ_0008835, bta_circ_0006815, bta_circ_0004158, bta_circ_0006812, bta_circ_0006152, bta_circ_0006807, bta_circ_0006806, bta_circ_0008914			
